# Supplementary material for: Moral judgment of objectionable online content: Reporting decisions and punishment preferences on social media
Source: PLoS One. 2024 Mar 25;19(3):e0300960. doi: 10.1371/journal.pone.0300960 (PMC10962817; doi:10.1371/journal.pone.0300960)
Supplement: S1 File — This file contains various supporting materials, methods and analyses, including, (1) Moral Images: This section details the images used in the Judgment and Punishment Tasks respectively, including the ‘Moral Wrongfulness’ ratings for each individual image (S1 and S2 Tables); (2) Questionnaires: This section presents each question that participants completed; and (3) Exploratory Analyses: This section includes exploratory graphs on participant responses as function of political orientation (S3 Table and S2 Fig), and current country of residence (S4 Table, S3 and S4 Figs). (DOCX) [file pone.0300960.s001.docx]

**Supporting information**

1. **Moral Images:** This section details the images used in the Judgment and Punishment Tasks respectively, including the ‘Moral Wrongfulness’ ratings for each individual image (**S1 Table** and **S2 Table**).
2. **Questionnaires:** This section presents each question that participants completed.
3. **Exploratory Analyses:** This section includes exploratory graphs on participants’ responses as function of political orientation (**S3 Table** and **S2 Fig**), and current country of residence (**S4 Table**, **S3 Fig** and **S4 Fig**).

**I. Images**

The Social-Moral Images Database (Crone et al., 2018) contains 2941 images, representing a wide range of morally (and affectively) positively, negative, and neutral content. Normative ratings from 1812 participants were available on eight dimensions, including the level of Moral Wrongness (1=*immoral/blameworthy*; 5=*moral/praiseworthy*) depicted in the image.

Upon thorough review of the 2941 images, the first author selected 66 images according to ‘Moral Wrongness’ rating, specifically 22 images with high scores on this scale (labelled as “morally positive”; *M_positive_*=4.65, *SD*_positive_=0.07); 22 images with low ‘Moral Wrongness’ ratings (labelled these as “morally negative”; *M_negative_*=1.27, *SD*_negative_=0.09), and 22 images falling in the middle of the range (labelled as “morally neutral”; *M_neutral_*=3, *SD*_neutral_=0). The selection of “morally negative” images specifically excluded images which could be considered as extremely graphic, sexually explicit and/or shocking (for example, naked or mutilated bodies). The images were presented in a random order to all participants in the Judgment Task. The 66 images are presented in Table S1.

**S1 Table.** **Images used in the Judgment Task.**

| **Image name** | **‘Moral Wrongness’** | **Category label** |
| --- | --- | --- |
| b15_p317_6 | 4,778 | morally positive |
| b15_p321_1 | 4,733 | morally positive |
| b15_p415_1 | 4,72 | morally positive |
| b2_p20_14 | 4,714 | morally positive |
| b14_p277_10 | 4,7 | morally positive |
| b14_p275_19 | 4,697 | morally positive |
| b4_p54_11 | 4,697 | morally positive |
| b11_p169_10 | 4,69 | morally positive |
| b15_p463_4 | 4,68 | morally positive |
| b10_p137_15 | 4,676 | morally positive |
| b10_p140_20 | 4,65 | morally positive |
| b11_p171_10 | 4,633 | morally positive |
| b15_p335_18 | 4,63 | morally positive |
| b15_p393_17 | 4,618 | morally positive |
| b4_p67_1 | 4,6 | morally positive |
| b2_p18_11 | 4,6 | morally positive |
| b11_p178_5 | 4,593 | morally positive |
| b13_p248_15 | 4,585 | morally positive |
| b15_p444_19 | 4,576 | morally positive |
| b15_p475_16 | 4,562 | morally positive |
| b15_p473_6 | 4,545 | morally positive |
| b15_p380_5 | 4,536 | morally positive |
| b999_p495_15 | 3 | morally neutral |
| b999_p491_15 | 3 | morally neutral |
| b15_p461_14 | 3 | morally neutral |
| b999_p479_12 | 3 | morally neutral |
| b999_p479_13 | 3 | morally neutral |
| b999_p482_20 | 3 | morally neutral |
| b15_p384_8 | 3 | morally neutral |
| b15_p450_12 | 3 | morally neutral |
| b13_p228_10 | 3 | morally neutral |
| b13_p233_20 | 3 | morally neutral |
| b14_p267_14 | 3 | morally neutral |
| b14_p271_15 | 3 | morally neutral |
| b15_p350_17 | 3 | morally neutral |
| b8_p119_18 | 3 | morally neutral |
| b11_p169_12 | 3 | morally neutral |
| b11_p178_16 | 3 | morally neutral |
| b5_p80_18 | 3 | morally neutral |
| b6_p87_19 | 3 | morally neutral |
| b6_p88_8 | 3 | morally neutral |
| b7_p101_6 | 3 | morally neutral |
| b8_p109_12 | 3 | morally neutral |
| b2_p16_20 | 3 | morally neutral |
| b15_p363_1 | 1,393 | morally negative |
| b11_p164_16 | 1,393 | morally negative |
| b999_p477_17 | 1,389 | morally negative |
| b15_p409_3 | 1,37 | morally negative |
| b11_p173_7 | 1,357 | morally negative |
| b11_p178_14 | 1,345 | morally negative |
| b4_p54_19 | 1,345 | morally negative |
| b15_p388_1 | 1,314 | morally negative |
| b15_p409_4 | 1,297 | morally negative |
| b5_p82_6 | 1,288 | morally negative |
| b15_p332_20 | 1,273 | morally negative |
| b10_p136_15 | 1,265 | morally negative |
| b15_p355_20 | 1,259 | morally negative |
| b4_p57_20 | 1,25 | morally negative |
| b10_p136_11 | 1,233 | morally negative |
| b11_p167_16 | 1,207 | morally negative |
| b2_p20_18 | 1,194 | morally negative |
| b15_p335_3 | 1,172 | morally negative |
| b11_p167_19 | 1,172 | morally negative |
| b2_p27_2 | 1,145 | morally negative |
| b2_p28_8 | 1,111 | morally negative |
| b999_p478_2 | 1,094 | morally negative |

*Note.* Image names are taken from the SMID database (Crone et al., 2018) and are listed here in descending order of Moral Wrongness, per category label.

**S2 Table.** **Images used in the Punishment Task.**

| **Image name** | **‘Moral Wrongness’** | **Category label** |
| --- | --- | --- |
| b14_p275_19 | 4,697 | morally positive |
| b11_p169_10 | 4,69 | morally positive |
| b15_p463_4 | 4,68 | morally positive |
| b10_p137_15 | 4,676 | morally positive |
| b10_p140_20 | 4,65 | morally positive |
| b15_p393_17 | 4,618 | morally positive |
| b11_p178_5 | 4,593 | morally positive |
| b13_p248_15 | 4,585 | morally positive |
| b15_p444_19 | 4,576 | morally positive |
| b15_p475_16 | 4,562 | morally positive |
| b15_p473_6 | 4,545 | morally positive |
| b15_p380_5 | 4,536 | morally positive |
| b999_p495_15 | 3 | morally neutral |
| b999_p491_15 | 3 | morally neutral |
| b15_p461_14 | 3 | morally neutral |
| b999_p479_12 | 3 | morally neutral |
| b999_p479_13 | 3 | morally neutral |
| b999_p482_20 | 3 | morally neutral |
| b8_p119_18 | 3 | morally neutral |
| b11_p169_12 | 3 | morally neutral |
| b11_p178_16 | 3 | morally neutral |
| b5_p80_18 | 3 | morally neutral |
| b6_p87_19 | 3 | morally neutral |
| b6_p88_8 | 3 | morally neutral |
| b999_p477_17 | 1,389 | morally negative |
| b15_p409_3 | 1,37 | morally negative |
| b4_p54_19 | 1,345 | morally negative |
| b15_p409_4 | 1,297 | morally negative |
| b15_p332_20 | 1,273 | morally negative |
| b10_p136_15 | 1,265 | morally negative |
| b10_p136_11 | 1,233 | morally negative |
| b2_p20_18 | 1,194 | morally negative |
| b15_p335_3 | 1,172 | morally negative |
| b2_p27_2 | 1,145 | morally negative |
| b2_p28_8 | 1,111 | morally negative |
| b999_p478_2 | 1,094 | morally negative |

*Note.* Image names are taken from the SMID database (Crone et al., 2018) and are listed here in descending order of Moral Wrongness, per category label.

**II. Questionnaires**

**QUESTION TO MEASURE EMPATHIC TENDENCIES:**

Derived from Interpersonal Reactivity Index (Davis, 1980)

The following statements are about your thoughts and feelings in a variety of situations. For each item, indicate how well it describes you by choosing the appropriate letter on the scale: A, B, C, D, or E. Answer as honestly as you can.

**ANSWER SCALE:
A = DOES NOT DESCRIBE ME VERY WELL, 
E = DESCRIBES ME VERY WELL**

|  | **A** | **B** | **C** | **D** | **E** |
| --- | --- | --- | --- | --- | --- |
| 1. I often have tender, concerned feelings for people less fortunate than me. |  |  |  |  |  |
| 2. I sometimes find it difficult to see things from the "other guy's" point of view. |  |  |  |  |  |
| 3. Sometimes I don't feel very sorry for other people when they are having problems. |  |  |  |  |  |
| 4. In emergency situations, I feel apprehensive and ill-at-ease. |  |  |  |  |  |
| 5. I try to look at everybody's side of a disagreement before I make a decision. |  |  |  |  |  |
| 6. When I see someone being taken advantage of, I feel kind of protective towards them. |  |  |  |  |  |
| 7. I sometimes feel helpless when I am in the middle of a very emotional situation. |  |  |  |  |  |
| 8. I sometimes try to understand my friends better by imagining how things look from their perspective. |  |  |  |  |  |
| 9. When I see someone get hurt, I tend to remain calm. |  |  |  |  |  |
| 10. Other people's misfortunes do not usually disturb me a great deal. |  |  |  |  |  |
| 11. If I'm sure I'm right about something, I don't waste much time listening to other people's arguments. |  |  |  |  |  |
| 12. Being in a tense emotional situation scares me. |  |  |  |  |  |
| 13. When I see someone being treated unfairly, I sometimes don't feel very much pity for them. |  |  |  |  |  |
| 14. I am usually pretty effective in dealing with emergencies. |  |  |  |  |  |
| 15. I am often quite touched by things that I see happen. |  |  |  |  |  |
| 16. I believe that there are two sides to every question and try to look at them both. |  |  |  |  |  |
| 17. I would describe myself as a pretty soft-hearted person. |  |  |  |  |  |
| 18. I tend to lose control during emergencies. |  |  |  |  |  |
| 19. When I'm upset at someone, I usually try to "put myself in his shoes" for a while. |  |  |  |  |  |
| 20. When I see someone who badly needs help in an emergency, I go to pieces (i.e. so upset/nervous that I cannot function normally). |  |  |  |  |  |
| 21. Before criticizing somebody, I try to imagine how I would feel if I were in their place. |  |  |  |  |  |

**QUESTION TO MEASURE POLITICAL ORIENTATION:**

On a scale from **1: Very Liberal (left-leaning)** to **7: Very Conservative (right-leaning)**, how would you describe your political orientation?

| **Very Liberal** | 1234567 | **Very Conservative** |
| --- | --- | --- |

**QUESTION TO MEASURE SOCIAL MEDIA USAGE:**

The following questions are about how you usually use social media. This includes how you would describe your social media use over the last **6 (six)** months.
**How often do you use these social network platforms?**

|  | **Multiple times a day** | **About once a day** | **A few times a week** | **A few times a month** | **About once a month or less** | **Never** |
| --- | --- | --- | --- | --- | --- | --- |
| Youtube |  |  |  |  |  |  |
| Instagram |  |  |  |  |  |  |
| Tiktok |  |  |  |  |  |  |
| Twitter |  |  |  |  |  |  |
| Facebook |  |  |  |  |  |  |
| Discord |  |  |  |  |  |  |
| Twitch |  |  |  |  |  |  |
| Reddit |  |  |  |  |  |  |
| Snapchat |  |  |  |  |  |  |
| LinkedIn |  |  |  |  |  |  |
| Pinterest |  |  |  |  |  |  |
| 9gag |  |  |  |  |  |  |

**QUESTIONS TO MEASURE SOCIAL MEDIA TRUST:**

Please indicate the degree to which the sentence below captures your feelings about each of the social media platforms.
Please note: this question refers to your thoughts on **the platform itself** and **not** the users of the platform.

**"I trust this social media platform and believe that the people/company that run and manage it are honest."**

|  | **Strongly Disagree** | **Disagree** | **Undecided** | **Agree** | **Strongly Agree** |
| --- | --- | --- | --- | --- | --- |
| Youtube |  |  |  |  |  |
| Instagram |  |  |  |  |  |
| Tiktok |  |  |  |  |  |
| Twitter |  |  |  |  |  |
| Facebook |  |  |  |  |  |
| Discord |  |  |  |  |  |
| Twitch |  |  |  |  |  |
| Reddit |  |  |  |  |  |
| Snapchat |  |  |  |  |  |
| LinkedIn |  |  |  |  |  |
| Pinterest |  |  |  |  |  |
| 9gag |  |  |  |  |  |

Please indicate the degree to which the sentence below captures your feelings about the users of the social media platforms mentioned.

Please note: this question refers to your thoughts on **the users** and **not** the platform.

**"I trust the users of this platform and believe that those who use and share content on it are honest."**

|  | **Strongly Disagree** | **Disagree** | **Undecided** | **Agree** | **Strongly Agree** |
| --- | --- | --- | --- | --- | --- |
| Youtube |  |  |  |  |  |
| Instagram |  |  |  |  |  |
| Tiktok |  |  |  |  |  |
| Twitter |  |  |  |  |  |
| Facebook |  |  |  |  |  |
| Discord |  |  |  |  |  |
| Twitch |  |  |  |  |  |
| Reddit |  |  |  |  |  |
| Snapchat |  |  |  |  |  |
| LinkedIn |  |  |  |  |  |
| Pinterest |  |  |  |  |  |
| 9gag |  |  |  |  |  |

**QUESTION TO MEASURE SOCIAL MEDIA OPINION:**

Please indicate the response which most accurately captures your experience with inappropriate or harmful content on social media.

**1. I know how to report or 'flag' inappropriate or harmful content I see on see on social media.**

Strongly Disagree – Disagree – Undecided – Agree – Strongly Agree

**2. I feel a sense of responsibility to report or 'flag' inappropriate or harmful content I see on social media.**

Strongly Disagree – Disagree – Undecided – Agree – Strongly Agree

**3. I've seen content I would consider inappropriate or harmful on social media.**

Never – Very Rarely – Rarely – Occasionally – Very Frequently – Always

**4. I report or 'flag' content I consider inappropriate or harmful on social media.**

Never – Very Rarely – Rarely – Occasionally – Very Frequently – Always

**5. I think those that post content I would consider inappropriate or harmful on social media should be punished.**

Strongly Disagree – Disagree – Undecided – Agree – Strongly Agree

**III. Exploratory Analyses**

**POLITICAL ORIENTATION**

**S3 Table. Political Orientation.** “On a scale from 1: Very Liberal (left-leaning) to 7: Very Conservative (right-leaning), how would you describe your political orientation?”

| ***Political Orientation*** | ***n(%)*** |
| --- | --- |
| *1* (Very Liberal) | 38 (12.93%) |
| 2 | 91 (30.95%) |
| 3 | 74 (25.17%) |
| 4 | 58 (19.73%) |
| 5 | 25 (8.50%) |
| 6 | 5 (1.70%) |
| 7 (Very Conservative) | 3 (1.02%) |

*Note.* Participants self-described as liberal (1 – 3) = 203 (69%); Participants self-described as equally liberal and conservative (4) = 58 (19.7%); Participants self-described as conservative (5 – 7) = 33 (11.2%).

**Figure S2. Total Reporting Rate in Judgment Task as function of participant (self-reported) Political Orientation (1=‘Very Liberal’; 7=’Very Conservative’).**

**COUNTRY OF RESIDENCE**

**S4 Table.** Number of Participants per Country of Residence

| **Country of Residence** | **Count** | **Percentage of Sample** |
| --- | --- | --- |
| Austria | 3 | 1.02% |
| Belgium | 4 | 1.36% |
| Czech Republic | 2 | 0.68% |
| Denmark | 1 | 0.34% |
| Estonia | 5 | 1.70% |
| Finland | 1 | 0.34% |
| France | 6 | 2.04% |
| Germany | 40 | 13.61% |
| Greece | 10 | 3.40% |
| Hungary | 6 | 2.04% |
| Ireland | 8 | 2.72% |
| Italy | 22 | 7.48% |
| Latvia | 4 | 1.36% |
| Luxembourg | 1 | 0.34% |
| Netherlands | 5 | 1.70% |
| Poland | 64 | 21.77% |
| Portugal | 59 | 20.07% |
| Slovenia | 1 | 0.34% |
| Spain | 48 | 16.33% |
| Sweden | 3 | 1.02% |

***Note.*** *Count represents number of participants from the study sample of n=294.*

******

**Figure S3. Mean Total Reporting Rate in Task 1 (Judgment Task) by Participant’s Current Country of Residence.** Error bars represent standard error of mean.

******

**Figure S4. Mean Punishment in Task 2 (Punishment Task (0 – 30 days)) by Participant’s Current Country of Residence.** Error bars represent standard error of mean.
