# Supplementary material for: Moral judgment of objectionable online content: Reporting decisions and punishment preferences on social media
Source: PLoS One. 2024 Mar 25;19(3):e0300960. doi: 10.1371/journal.pone.0300960 (PMC10962817; doi:10.1371/journal.pone.0300960)
Supplement: Attachment — Submitted filename: Response to Reviewer.docx [file pone.0300960.s003.docx]

**Rebuttal Letter**

Journal Requirements:

Please review your reference list to ensure that it is complete and correct. If you have cited papers that have been retracted, please include the rationale for doing so in the manuscript text, or remove these references and replace them with relevant current references. Any changes to the reference list should be mentioned in the rebuttal letter that accompanies your revised manuscript. If you need to cite a retracted article, indicate the article’s retracted status in the References list and also include a citation and full reference for the retraction notice.

We thank the editor for this comment. We have ensured that none of the references cited in our manuscript have been retracted. In addition, we have ensured that each of the citations are edited according to PLoS ONE’s style/journal requirements. To ensure that our list is complete and concise, we have removed the following two references from the manuscript: *McLoughlin, K. L., Brady, W. J., & Crockett, M. J. (2021). The role of moral outrage in the spread of misinformation* (conference proceeding abstract, previously labelled as reference number 13 on lines 97 and 105,) and *Tucker, J. A., Guess, A., Barberá, P., Vaccari, C., Siegel, A., Sanovich, S., ... & Nyhan, B. (2018). Social media, political polarization, and political disinformation: A review of the scientific literature. Political polarization, and political disinformation: a review of the scientific literature (March 19, 2018)* (previously labelled as reference number 8 on line 61).

Reviewers' comments:

Review Comments to the Author

Please use the space provided to explain your answers to the questions above. You may also include additional comments for the author, including concerns about dual publication, research ethics, or publication ethics. (Please upload your review as an attachment if it exceeds 20,000 characters)

Reviewer #2: I have now read the revised manuscript and the authors' responses to the review comments. I think the comments and suggestions have been addressed adequately and I recommend accepting the manuscript for publication.

We sincerely appreciate the reviewers thorough evaluation of both the original and revised manuscript, as well as our responses to previous comments.
